# Cone-Beam Computed Tomography Evaluation of Maxillary Sinus Changes in Orthodontic Patients Treated With Extraction of Four First Premolars

**DOI:** 10.7759/cureus.65029

**Published:** 2024-07-21

**Authors:** Kaiyang Xue, Yuyan Tao, Dan Pan, Runze Wang, Yuyao Zhang, Shufang Du, Wen Liao

**Affiliations:** 1 Department of Orthodontics, State Key Laboratory of Oral Diseases & National Clinical Research Center for Oral Diseases, West China Hospital of Stomatology, Sichuan University, Chengdu, CHN

**Keywords:** premolar, orthodontic treatment, maxillary sinus, tooth extraction, cbct

## Abstract

The objective of this study was to assess alterations in maxillary sinus mucosa thickness and the distances between the apexes of specific teeth and the maxillary sinus base in adult patients undergoing orthodontic treatment with the extraction of four first premolars. Twenty-one adults, averaging 24.85 years of age, received orthodontic therapy involving the extraction of all four first premolars. Cone-beam computed tomography scans were conducted before and after treatment to evaluate changes. Notably, post-treatment scans revealed a significant increase (P= 0.044) in the distance between the apex of the second premolar and the maxillary sinus floor, with an average augmentation of 1.0141 millimeters. However, no notable alterations were detected in the distances between the apexes of other teeth and the maxillary sinus or in maxillary sinus mucosa thickness. These findings suggest that orthodontic treatment with the extraction of four first premolars may elevate the distance between the maxillary sinus floor and the second premolar apex, which provides a reference for risk assessment and surgical design of first premolar extraction during orthodontic treatment.

## Introduction

The maxillary sinus, the largest para-nasal sinus located within the maxilla [1], is composed of six walls, including the anterior wall, posterior wall, superior wall, inferior wall, medial wall, and lateral wall [2]. The base of the maxillary sinus is formed by the alveolar process of the maxilla and part of the hard palate bone and is closely connected to the root of the maxillary posterior teeth. These roots are very close to or even extend to the base of the maxillary sinus [3]. When the tooth moves under the orthodontic force during orthodontic treatment, its root tip may shift within the maxillary sinus cortical bone [4]. This process is achieved through the absorption of the anterior and lower bone structures, providing the necessary space for tooth movement. At the same time, there may be new bone deposition in the opposite direction, increasing bone density and strength, thereby maintaining the stability of the teeth and the integrity of the surrounding bone structure [5].

The root tips of the upper molars, premolars, and even posterior teeth are separated from the maxillary sinus by the alveolar bone or only covered by the mucous membrane of the maxillary sinus [6,7]. A large number of studies have shown that the root tip of the first premolar is closest to the base of the maxillary sinus and is closely related to the maxillary sinus [8,9]. According to relevant literature reports, removal of the first premolar tooth during orthodontic treatment will expand the maxillary sinus base downward, increasing maxillary sinus volume and decreasing the distance between the root of the posterior tooth and the sinus base [10]. This creates additional resistance that may alter the original design balance of the orthodontic treatment and may affect bone remodeling activity, affecting the normal pattern of bone remodeling, and may hinder the desired tooth movement to the desired position, thereby affecting the orthodontic outcome. Given the close relationship between the first premolar and the maxillary sinus, it is expected that the volume and mucosal thickness of the first premolar will change during orthodontic treatment, especially after all four first premolars are removed.

Due to the subcortical bone in the maxillary sinus floor, orthodontic tooth movement may be impeded. When a tooth root protrudes into the maxillary sinus during orthodontic treatment, there is a risk of root resorption and tipping [11]. Therefore, it is crucial to understand the anatomical relationship between the maxillary sinus floor and the maxillary tooth roots for the proper formulation of an orthodontic treatment plan. Additionally, it is unclear whether the anatomical structure and physiology of the maxillary sinus change during orthodontic tooth extraction. Mucosal thickening is a common inflammatory response of the maxillary sinus, considered pathological when exceeding 2 mm [12]. A moderate correlation exists between maxillary sinus mucosal thickness and inflammation [13]. Thus, it is important to assess the potential impact of orthodontic tooth extraction on maxillary sinus mucosal thickness. This study aims to use cone-beam computed tomography (CBCT) to measure changes in maxillary sinus mucosal thickness and the distance between the apices of the second premolars, first molars, and second molars and the base of the maxillary sinus before and after extraction of the four first premolars during orthodontic treatment to provide valuable insights for risk assessment and surgical planning in first premolar traction during orthodontic treatment.

## Materials and methods

Subjects

This retrospective analysis focuses on orthodontic treatment conducted at the Department of Orthodontics, the West China Hospital of Stomatology, Sichuan University in Chengdu, China. The study period lasted from April 2016 to June 2023. Patient data were collected from the hospital's medical record database and carefully reviewed. The researchers examined the recorded diagnoses and treatment characteristics of the patients. Using G*Power 3.1.3, we performed a power analysis to determine the required sample size. The analysis indicated that a minimum number of 19 participants is necessary to achieve 95% power at a significance level of 0.05, with an effect size of 0.8. The research samples were selected based on specific inclusion criteria, which are presented below.

Inclusion Criteria

Patients were required to have undergone CBCT imaging before and after orthodontic treatment, with a maximum interval of two weeks. CBCT images were expected to encompass cranial and maxillofacial skeletal structures from the orbit to the mandible, ensuring clarity and absence of artifacts, and including complete visualization of the maxillary sinuses. Patients were to be over 18 years old, possessing intact dentition from central incisors to second molars, without supernumerary teeth, dental defects, or metallic restorations. Patients must have undergone extraction of the four first premolars during the orthodontic intervention. Patients must have utilized fixed orthodontic appliances during their treatment. Moderate anchorage preservation was necessary during space closure procedures. Post treatment, patients should have achieved complete space closure and attained satisfactory functional occlusion.

Exclusion Criteria

Exclusion criteria included patients with clinically diagnosed maxillary sinusitis, history of other maxillary sinus diseases, craniofacial anomalies, or significant periodontal concerns. Patients who had undergone prior maxillofacial surgery. CBCT images that did not fully cover bilateral maxillary sinuses.

The CBCT images utilized in this study were obtained using a 3D Accuitomo CBCT machine (3D Accuitomo, Morita Group, Saitama, Japan), following the manufacturer's recommendations (140 × 100 mm FOV, 85 kV, 4.0 mA, and 360° rotation). The voxel size of the images was 125 µm. Subsequently, the CBCT data were saved in DICOM multifile format. All patients received treatment with the Damon Q self-ligating orthodontic appliance (Damon Q, Ormco Corporation, Orange, CA). The study was conducted in compliance with the principles outlined in the Declaration of Helsinki.

The research has been approved by the Ethics Committee of West China Hospital of Stomatology, Sichuan University (WCHSIRB-CT-2021-479).

Maxillary sinus analysis

**Figure 1:**
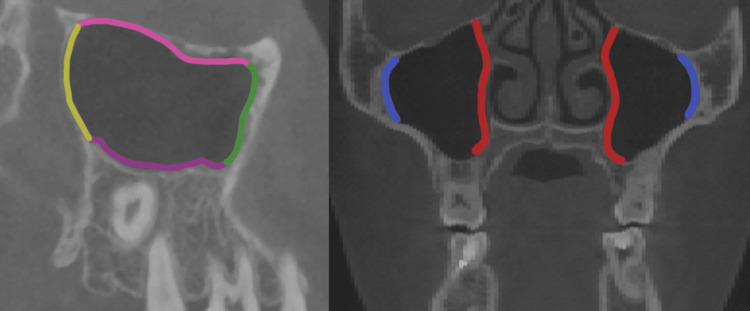
The delineation of the maxillary sinus walls is as follows: red denotes the lateral wall, blue represents the medial wall, yellow indicates the anterior wall, green signifies the posterior wall, purple outlines the superior wall, and pink highlights the inferior wall.

**Figure 2:**
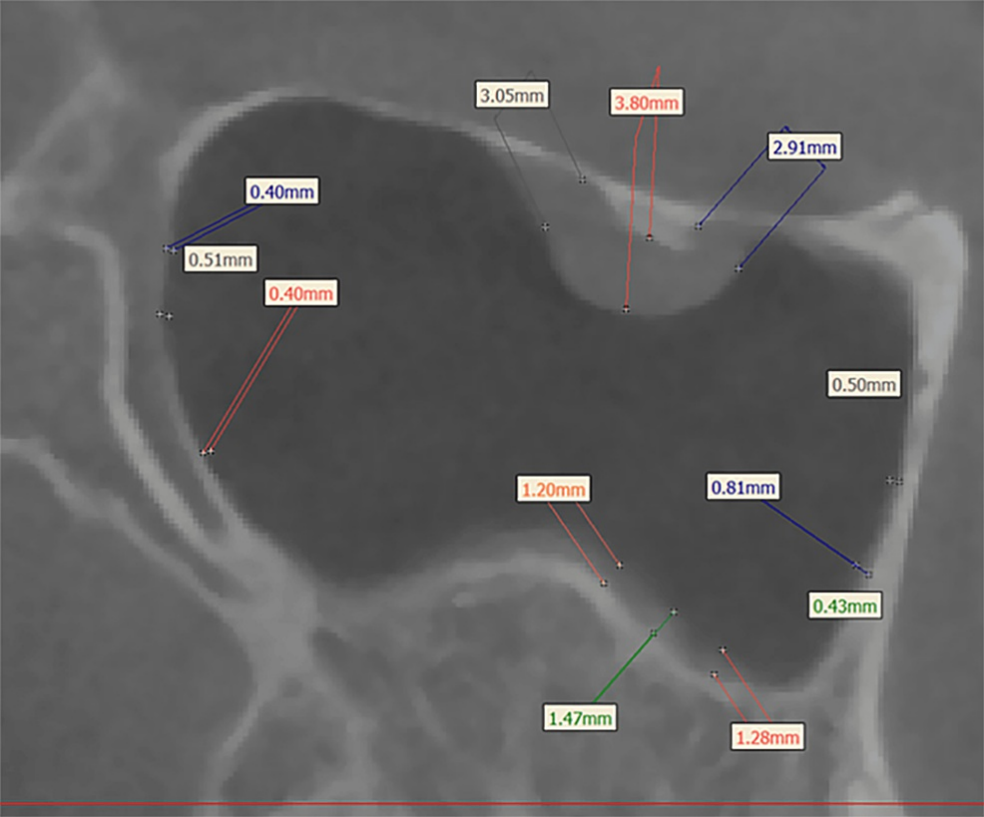
In the maxillary sinus, measure the thickness of the mucous membrane at three thickest points on each wall, and take the largest value as the final maximum mucosal thickness.

**Figure 3:**
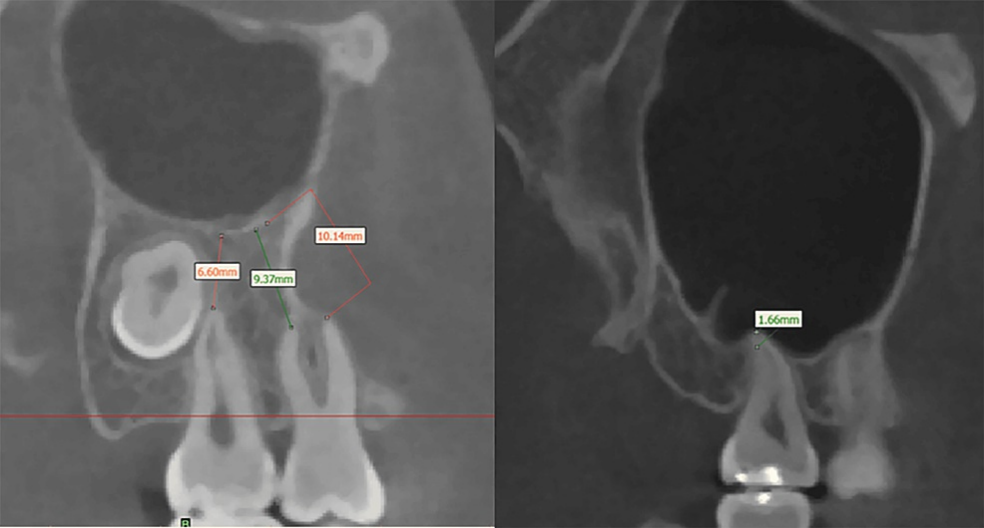
Measurement of the distance between the tooth root and the maxillary sinus in two different scenarios.

We employed Dolphin software (version 11.8; Dolphin Imaging and Management Solutions; Chatsworth, CA) to import both pre-treatment (T1) and post-treatment (T2) DICOM data. Within the CBCT images of the maxillary sinus, sagittal images delineated the anterior, posterior, superior, and inferior walls, while coronal images illustrated the medial and lateral walls (Figure 1). Utilizing Mimics software (version 19.0; Materialise, Leuven, Belgium), we assessed the maxillary sinus mucosa thickness and the distance from dental roots to the sinus floor. The sagittal images of the anterior, posterior, superior, and inferior walls, coupled with coronal images of the medial and lateral walls, facilitated the measurement of the thickest mucosal areas. Mucosal thickness was determined by measuring at three points on each wall, with the maximum value selected as the final thickness (Figure 2). In the sagittal plane images, we measured distances between the mesiobuccal (MB), distobuccal (DB), and palatal (Pa) root apices of the maxillary second premolars and molars and the lower sinus wall. When roots did not contact the sinus wall, we measured the shortest distance from the apex to the sinus floor, considered a positive value. Conversely, if the root apex extended into the sinus, the shortest distance from the apex to the sinus floor was measured as a negative value (Figure 3). All measurements were conducted by an experienced orthodontist proficient in CBCT assessments.

Statistical analysis

The statistical analysis for this study utilized the SPSS software (version 22.0; IBM Corp., Armonk, NY). To assess the level of agreement, intraobserver agreements were measured using the intraclass correlation coefficient (ICC). To examine the changes before and after treatment, a paired t-test and the Wilcoxon signed-rank test were employed.

## Results

A total of 42 samples from the maxillary sinus were collected from 21 patients with a mean age of 24.85 years who had undergone extraction of their maxillary and mandibular bilateral first premolars. The average time interval between the pre- and post-treatment records was 29.23 months.

The reliability of intraobserver measurements was found to be good, with ICC values ranging from 0.832 to 0.999.

Concerning the thickness of the maxillary sinus mucosa, measurements of the anterior, posterior, medial, lateral, superior, and inferior mucosal thicknesses revealed no statistically significant differences before and after treatment (P > 0.05) (Table 1).

**Table 1:** Evaluation of T2-T1 changes in mucosal thickness of each wall of the maxillary sinus.

Variables	Mean	SD	P-value
T2-T1 change of thickness of the anterior wall mucosa of the maxillary sinus	-0.0224	0.3561	0.950
T2-T1 change of thickness of the posterior wall mucosa of the maxillary sinus	-0.0677	0.0928	0.467
T2-T1 change of thickness of the superior wall mucosa of the maxillary sinus	0.2654	0.3539	0.456
T2-T1 change of thickness of the inferior wall mucosa of the maxillary sinus	-0.3654	0.6602	0.582
T2-T1 change of thickness of the medial wall mucosa of the maxillary sinus	0.2308	0.5890	0.696
T2-T1 change of thickness of the lateral wall mucosa of the maxillary sinus	-0.3072	0.7568	0.686

In terms of the distance between the roots of maxillary premolars and molars and the floor of the maxillary sinus, a notable finding emerged: post-treatment analysis indicated that the second premolar was situated further from the sinus floor (P = 0.044) with an average increase of 1.0141 millimeters. There was no statistically significant change in the distance between the MB, DB, and Pa root apices of the first and second molars from the floor of the maxillary sinus before and after treatment (Table 2).

**Table 2:** Evaluation of T2-T1 changes in distance of molar and premolar roots to the inferior sinus wall.

Variables	Mean	SD	P-value
T2-T1 change of distance from the apex of the second premolar on the maxilla	1.0141	0.4968	0.044
T2-T1 change of distance from the apex of the mesiobuccal root of the first molar on the maxilla	-0.2396	0.5631	0.672
T2-T1 change of distance from the apex of the distobuccal root of the first molar on the maxilla	-0.0755	0.4688	0.872
T2-T1 change of distance from the apex of the palatal root of the first molar on the maxilla	-0.4243	0.6636	0.524
T2-T1 change of distance from the apex of the mesiobuccal root of the second molar on the maxilla	-0.1231	0.5589	0.826
T2-T1 change of distance from the apex of the distobuccal root of the second molar on the maxilla	0.0582	0.5822	0.921
T2-T1 change of distance from the apex of the palatal root of the second molar on the maxilla	-0.3330	0.6104	0.587

## Discussion

In this study, 42 maxillary sinus specimens collected after extraction of bilateral maxillary first premolars in 21 patients were analyzed in detail to evaluate the effects of extraction of four first premolars during orthodontic treatment on maxillary sinus mucosal thickness and the distance between the root and the maxillary sinus base wall. The CBCT imaging results showed no statistically significant changes in the thickness of maxillary sinus mucosa in the six walls before and after orthodontic treatment. Except for the second premolar, the distance between the root tip and the maxillary sinus base was not significantly changed.

In orthodontic treatment, the extraction of the first premolar is a common method to deal with crowded teeth or other dental deformities [8,14]. The position of the first premolar is close to the maxillary sinus, and its root tip is very close to the bottom of the maxillary sinus [15]. Therefore, when removing the first premolar during orthodontic treatment, doctors need to carefully consider whether the removal of the first premolar has a potential impact on the mucosa of the maxillary sinus.

Change of thickness of the mucosa of the maxillary sinus walls

This study showed that after the first premolar extraction, the thickness of maxillary sinus mucosa did not change before and after orthodontic treatment, which may be related to the following reasons. First, the force exerted by orthodontic treatment mainly affects the position of alveolar bone and teeth [16,17] and has little direct impact on maxillary sinus mucosa. Tooth movement and alveolar bone remodeling do not cause significant thickness changes in maxillary sinus mucosa [15,18]. Secondly, there may be a certain biomechanical relationship between the mucosa of the maxillary sinus and the floor wall of the maxillary sinus [19]. Even if the alveolar bone is reconstructed, the thickness of the mucosa may remain stable through some mechanism. In addition to the above reasons, the maxillary sinus, as an air chamber, may have a certain stability and adaptability of its internal mucosa [19-21], and the thickness of the mucosa can remain unchanged during alveolar bone remodeling caused by tooth extraction and orthodontic treatment. This finding has important implications for orthodontic clinical practice, as it suggests that the removal of the first premolar may not cause irreversible damage to the maxillary sinus mucosa, thus reducing the concern of tooth extraction in orthodontic treatment.

Change of distance from the apex of premolars and molars on the maxillary sinus

Additionally, we observed that after orthodontic treatment, the average distance between the root tip of the second premolar and the floor of the maxillary sinus increased by 1.0141 mm. This is an interesting finding as it may indicate that after tooth extraction, there has been resorption of the alveolar bone, which could lead to a decrease in the height of the alveolar bone, thereby increasing the relative distance between the floor of the maxillary sinus and the root tip. This phenomenon is considered to be related to two factors: (1) the mechanical forces applied during orthodontic treatment promote the resorption and reconstruction of alveolar bone, which may cause the root tip to move into the space previously occupied by the tooth, thereby increasing the distance between the root tip of the second premolar and the floor of the maxillary sinus. (2) During orthodontic treatment, periodontal tissues respond to mechanical forces with a series of adaptive changes, leading to a reduction in the height of the alveolar bone. Since the second premolar is adjacent to the extracted first premolar, after the first premolar is removed, to close the space created by the extraction, the adjacent tooth (such as the second premolar) may move, and its root tip may tilt toward the extraction area, resulting in an increased distance from the maxillary sinus base wall [22]. In addition, after tooth extraction, the alveolar bone will undergo a physiologic reconstruction process. Such remodeling usually includes bone resorption and new bone formation in the tooth extraction area [23,24], which may lead to changes in alveolar bone height and further increase the distance between the root tip of the second anterior molar and the maxillary sinus base wall. Besides, after tooth extraction, the periodontal ligament undergoes a remodeling process [25], and the fibers of the periodontal ligament may be rearranged to adapt to the new tooth arrangement and occlusal relationship. This adaptive change may affect the position of the teeth and the root tip's relative position to the maxillary sinus base wall, resulting in an increased distance between the root tip of the second anterior molar and the maxillary sinus base wall. Furthermore, compared to traditional brackets, using Damon self-ligating brackets during treatment will more noticeably cause expansion of the upper dental arch, especially in the premolar region [26,27], while also causing buccal inclination of the teeth [28], which may also be a reason for this change. However, we did not observe a statistically significant change in the distance between the root tips of the first and second molars and the floor wall of the maxillary sinus, which may be related to the direction of tooth movement and the anchoring technique used in orthodontic treatment.

Ciğerim et al. [29] studied the changes in maxillary sinus mucosal thickness in orthodontic patients with maxillary molar distalization and found that compared to untreated individuals, undergoing molar distalization orthodontic treatment can reduce changes in the mucosal thickness of the floor of the maxillary sinus. This is inconsistent with our research results, possibly due to different orthodontic treatment methods and the extraction of the first premolar. However, no statistically significant thickening was found in the mucosa of other maxillary sinus walls, which is consistent with our research results. Al-Worafi et al. [30] studied patients undergoing molar distalization using clear aligners and found that all the molar roots moved toward the maxillary sinus, especially the mesiobuccal root of the upper second molar protruding into the sinus. However, our research results show that, on average, most of the roots of the maxillary molars moved toward the maxillary sinus after treatment, but no statistically significant changes were found. This may be due to the treatment methods, different appliances used, and the extraction of the first premolar.

This study provides new insights into the relationship between teeth and the maxillary sinus during orthodontic extraction, reducing concerns about tooth intrusion into the maxillary sinus during the orthodontic extraction process, which could hinder tooth movement and cause a series of complications or thickening of the maxillary sinus mucosa. These data fill the theoretical gap regarding whether the anatomical structure of the maxillary sinus changes during orthodontic extraction, laying the foundation for further in-depth investigation.

Although our study provides valuable insights, there are some limitations to consider. First, our sample size is relatively small, which may limit the general applicability of the results. Second, our study is a cross-sectional study and cannot determine cause and effect. Future studies should consider conducting multi-center, large-sample size prospective studies to further validate our findings. In addition, future studies should explore differences in the impact of different orthodontic treatment options on the maxillary sinus and consider individual differences in patients. Finally, long-term follow-up studies are also necessary to assess the long-term effects of extraction and orthodontic treatments on the maxillary sinuses.

## Conclusions

In summary, our research provides insights into the impact of removing the first premolar during orthodontic treatment on the maxillary sinus. Although our results indicate that tooth extraction does not have a significant effect on mucosal thickness, we found that the apices of the second premolars are positioned further away from the floor of the maxillary sinus after the orthodontic treatment involving the removal of the first premolar. The impact on the distance between the tooth roots and the maxillary sinus wall still requires further exploration in future studies. With these findings, we can provide more accurate information for clinical decision-making regarding orthodontic treatment and offer better outcomes for patients.
